# Automatic calculation of patient size metrics in computed tomography: What level of computational accuracy do we need?

**DOI:** 10.1002/acm2.12240

**Published:** 2017-12-19

**Authors:** Sandra Sarmento, Bruno Mendes, Margarida Gouvêa

**Affiliations:** ^1^ Medical Physics, Radiobiology and Radiation Protection Group IPO Porto Research Center (CI‐IPOP) Portuguese Oncology Institute of Porto (IPO Porto) Porto Portugal; ^2^ Medical Physics Department Portuguese Oncology Institute of Porto (IPO Porto) Porto Portugal; ^3^ Radiology Department Portuguese Oncology Institute of Porto (IPO Porto) Porto Portugal

**Keywords:** computed tomography, examination doses, patient size metrics, water equivalent diameter

## Abstract

**Objectives:**

To compare the effectiveness of two different patient size metrics based on water equivalent diameter (*D*_w_), the mid‐scan water equivalent diameter *D*_w_c_, and the mean (average) water equivalent diameter in the imaged region, *D*_w_ave_, for automatic detection of accidental changes in computed tomography (CT) acquisition protocols.

**Methods:**

Patient biometric data (height and weight) were available from a previous survey for 80 adult chest examinations, and 119 adult single‐acquisition chest–abdomen–pelvis (CAP) examinations for two 16 slice scanners (GE LightSpeed and Toshiba Aquilion RXL) equipped with automatic tube current modulation (ATCM). *D*_w_c_ and *D*_w_ave_ were calculated from the archived CT images. Size‐specific dose estimates (SSDE) were obtained from volume CT dose index (CTDI_vol_), using the conversion factors for a patient diameter of *D*_w_c_.

**Results:**

CTDI_vol_ and SSDE correlate better with *D*_w_ave_ than with *D*_w_c_. *R*‐squared values of linear fits to CTDI_vol_ of CAP examinations were 0.81–0.89 for *D*_w_c_ and 0.93–0.94 for *D*_w_ave_ (SSDE: 0.69–080 for *D*_w_c_, 0.87–0.92 for *D*_w_ave_). Percentage differences between *D*_w_c_ and *D*_w_ave_ were −4 ± 4% for chest and +5 ± 4% for CAP examinations (in % of *D*_w_ave_). However, small *D*_w_ variations translated as larger variations in CTDI_vol_ for these ATCM systems (e.g., a 24% increase in *D*_w_ doubled CTDI_vol_). The dependence of CTDI_vol_ on *D*_w_ave_ was similar for chest and CAP examinations performed with similar ATCM parameters, while use of *D*_w_c_ resulted in a clear separation of the same data according to examination type. Maximum *D*_w_ variation in the imaged region was 5.6 ± 1.6 cm for chest and 6.5 ± 1.4 cm for CAP examinations.

**Conclusions:**

*D*_w_ave_ is a better metric than *D*_w_c_ for binning similar‐sized patients in dose comparison studies, despite the additional computational effort required for its calculation Therefore, when implementing automatic determination of *D*_w_ for SSDE calculations, automatic calculation of *D*_w_ave_ should be considered.

## 1 INTRODUCTION

Computed tomography (CT) is a powerful diagnostic tool, but CT imaging protocols should be optimized to minimize radiation exposure. Diagnostic reference levels (DRLs) have been a powerful tool in dose optimization, by establishing typical values of volume CT Dose Index (CTDI_vol_) or dose‐length product (DLP), for certain types of examinations performed on standard‐sized (70 ± 3 kg) patients.^1^, ^2^, ^3^, ^4^

Modern CT scanners are equipped with automatic tube current modulation (ATCM), which adjusts tube current according to patient size and anatomical region, based on parameters set by the user.^5^ The functioning of an ATCM system and the conditions to be set depend on the scanner manufacturer. For GE and Toshiba systems, three parameters must be specified: an “image quality index” related to image noise, and the minimum and maximum values of tube current, *I*_min_ and *I*_max_. An adequate value of *I*_max_ avoids unnecessary dose escalation in large or obese patients. *I*_min_ is equally important to prevent excessive noise in smaller patients, particularly in low attenuation regions such as the lungs.^6^, ^7^ GE defines a parameter called noise index (NI) to specify the noise level, while Toshiba uses the standard deviation (SD).^8^, ^9^

ATCM systems have some limitations, and pediatric patients need separate imaging protocols, with different parameters (such as lower kV and lower *I*_min_). The range of sizes in pediatric patients is immense, from babies to adolescents, and different protocols should be used according to child size/age.^8^, ^10^, ^11^ Only adult patients will be considered in the present work.

Optimization of ATCM settings is a time‐consuming process involving a radiologist who assesses image quality after each acquisition. This is usually done for only a few patients. If the results are considered acceptable, the protocol is implemented in a provisional fashion. Postacquisition assessment of examination doses and image quality is continued for some time, to confirm that settings are optimized for all patient sizes.^7^ In this context, it is useful to have reference dose levels for different‐sized adult patients. The American Association of Physicists in Medicine (AAPM) lists approximate reference values for different weight ranges in some typical examination protocols.^12^, ^13^

Reliance on ATCM systems increases the potential detriment of nonoptimized settings and accidental changes to previously optimized protocols, as follows. An accidental increase in target image noise will result in a degradation of image quality for all patients, which should be quickly detected by radiologists. However, a decrease in target image noise will increase examination doses and image quality for smaller adults, while scanner output for large patients is limited by *I*_max_. Likewise, an unnecessarily high value of *I*_min_ increases examination doses for small adults, with no degradation of image quality. Both situations lead to saturation of the tube current (at *I*_max_ or *I*_min_) for an increased number of patients,^7^ but this may go unnoticed under a heavy workload, or be dismissed through overconfidence in the automated system.

With the introduction of PACS (Picture Archiving and Communication Systems) in radiology, several vendors have developed radiation dose index monitoring (RDIM) software, which collects dosimetric information from imaging studies and stores it in a relational database.^14^ RDIM systems are a powerful tool to identify accidental changes and outliers, and determine where optimization is needed. However, patient biometric data (height and weight) are not usually available in PACS. Therefore, an accidental change which affects only small adults is hard to recognize quickly, because individual examination doses are still in the expected range (e.g., a 50‐kg adult imaged with a CTDI_vol_ adequate for a 90‐kg patient). Naturally, dosimetric data from thousands of examinations will include patients of all sizes and can be compared between different institutions and scanners. But this is population‐dependent and also impractical for quick detection of changes and nonoptimized protocols.

The AAPM Task Group 204 proposed the use of size‐specific dose estimates (SSDE) for patient dose comparisons. SSDE is an estimate of patient dose at the center of the imaged region, obtained from CTDI_vol_ using conversion factors f(*D*_eff_) related to the effective diameter of the patient, calculated from the measured anteroposterior (AP) and lateral (LAT) patient dimensions, *D*_eff_ = √(AP∙LAT).^15^ To improve the calculation of SSDE by taking into account patient attenuation, the AAPM Task Group 220 proposed describing patient size in terms of water equivalent diameter (*D*_w_). TG220 also suggested that *D*_w_ could be calculated automatically by the CT scanner for all patients, with no user intervention, and the results stored in the DICOM header of CT images.^16^ An automatically calculated *D*_w_ would allow binning of similar‐sized patients in RDIM databases for comparison of examination doses. SSDE values for adults may vary with patient size, depending on the ATCM system.^17^

Leng et al. have shown that SSDE can be calculated with less than 10% error using the examination CTDI_vol_ and the value of *D*_w_ obtained from the mid‐scan CT slice (*D*_w_c_).^16^ However, values of *D*_w_ along the imaged region, *D*_w_(*z*), may be useful for estimating organ doses.^16^, ^18^

Obtaining *D*_w_(*z*) values requires longer computational times, but it also allows calculation of the mean value (average) of *D*_w_(*z*), *D*_w_ave_. As the response of ATCM systems is based on patient attenuation, *D*_w_ave_ is the quantity more closely related to the examination's mean CTDI_vol._ 19Anam et al. recently reported on the implementation of automatic contouring for calculation of *D*_w_, and showed that *D*_w_ave_ could be obtained with reasonable accuracy from only nine images, for head and thorax examinations.^20^ This is still nine times the computational effort required for calculating *D*_w_c_. Differences between *D*_w_c_ and *D*_w_ave_ were found to be less than 10%,^20^ which agrees well with data reported by other authors.^21^

The aim of this study was to compare *D*_w_c_ and *D*_w_ave_ as patient size metrics, for the purpose of ATCM optimization and detection of accidental changes; and to determine whether the difference between the two is sufficient to justify the additional computational effort required to automatically determine *D*_w_ave_ in addition to *D*_w_c._ This study also assessed the interdependence of metrics and the feasibility of using *D*_w_ metrics in nonautomated scenarios, for retrospective comparison with older data.

## 2 MATERIALS AND METHODS

### 2.A Data collection

This study took advantage of existing biometric data, which had been collected during a routine internal survey, after confirmation by the radiologists that image quality was satisfactory. Patient biometric data (height and weight) were available for 80 chest and 119 single‐acquisition chest–abdomen–pelvis (CAP) examinations, performed in either of the two scanners available at our institution: a GE LightSpeed in use since 2011 (CT11) and the Toshiba Aquilion RXL acquired in March 2014 (CT14).

The available data, summarized in Tables 1 and 2, pertain only to adult patients (21–89 years, mean 62 years), because pediatric examinations use separate protocols. CAP and chest examinations were chosen because they are frequently performed and because the corresponding protocols are used as a basis for the examination protocols of more complex examinations, which are harder to optimize independently.

**Table 1:** Summary of patient data for chest examinations. Weight, height, and BMI are indicated as mean values, with the range in brackets

	# of Patients		Weight (kg)	Height (cm)	BMI (kg/m^2^)
CT11	31 (24 M; 7 F)		69 (45–105)	167 (150–183)	25 (16–38)
CT14	49 (27 M; 22 F)		69 (43–117)	164 (147–183)	25 (17–53)
Total	80	29 F	64 (43–117)	159 (147–175)	26 (17–53)
		51 M	72 (45–105)	169 (157–183)	25 (16–38)

**Table 2:** Summary of patient data for CAP examinations. Weight, height, and BMI are indicated as mean values, with the range in brackets

	No. of Patients		Weight (kg)	Height (cm)	BMI (kg/m^2^)
CT11	41 (22 M; 19 F)		65 (44–90)	163 (145–180)	25 (17–37)
CT14	78 (38 M; 40 F)		70 (43–104)	163 (144–185)	26 (17–34)
Total	119	59 F	65 (43–90)	159 (144–175)	26 (17–37)
		60 M	71 (45–104)	168 (145–185)	5 (17–34)

Both CT11 and CT14 are 16‐slice scanners, equipped with ATCM in both the longitudinal direction (*z*‐axis modulation) and the perpendicular plane (*xy* or angular modulation). The combination of these two is known as 3D modulation. Patients are randomly assigned to one scanner or the other, depending on equipment availability and internal logistics. Two orthogonal scout images (tube positions 0° and 90°) were acquired before each examination, in the order recommended by each manufacturer. The acquisition parameters are summarized in Table 3.

**Table 3:** Acquisition parameters used in both scanners

	kV	Collimation (mm)	Pitch	Time (s)/rot	Image quality index (NI/SD)	*I*_min_ (mA)	*I*_max_ (mA)
CT11	120	20 (16 × 1.25)	1.375	0.8	NI = 18	100	440
CT14	120	32 (16 × 2)	0.938	0.5	SD = 12.5	80/100^a^	500

a While data were being collected, the *I*_min_ value was changed for the CT14 scanner. This had little influence on patient doses. Therefore, only one dataset was considered. Image quality remained satisfactory to the radiologists in the department.

Proper functioning of the ATCM system and scanner indications of dosimetric parameters were checked at acceptance, and then annually, following the protocols and recommendations of the Spanish Medical Physics Society.^22^

To reduce patient dose in CT examinations, it is important to limit anatomical coverage (scan range) to the area of clinical concern.^7^ Appropriate restriction of anatomical coverage minimizes the scan length, whereas the optimization of ATCM parameters is reflected in the examination's mean CTDI_vol_. Both influence the dose‐length product (DLP). In this work, the examination CTDI_vol_ (mean CTDI_vol_ for the 32 cm diameter CTDI phantom) was chosen as the dosimetric parameter of interest and obtained from the dose summary archived in PACS.

### 2.B Retrospective data analysis using attenuation metrics

Reconstructed CT images can be used to calculate *D*_w_, provided the reconstruction kernel is linear and quantitative (not edge‐enhancing or otherwise nonlinear).^16^ The image series obtained with the SOFT (GE) and FC08 (Toshiba) reconstruction kernels were used for this study.

The field of view (FOV) used in chest and CAP examinations usually includes the outer contour of the patient. Visual observation of the CT images confirmed that, for the majority of the examinations, the whole contour of the skin was visible in the entire imaged region, except near the shoulders. Examinations where a large part of the patient's contour was outside the FOV were excluded from the dataset. These situations were too rare to justify a correction based on air border proportion, as suggested by Ikuta et al.^23^

According to the AAPM Task Group 220, the water equivalent diameter (*D*_w_) of an object is related to its water equivalent area (*A*_w_): $D_{w}=2\sqrt{A_{w}/\pi }$.^16^ If <CT>_ROI_ is the mean CT number in a ROI (region of interest) of area, A_ROI,_ containing the object, then *A*_w_ can be determined from a CT image using eq. (1), 16:

(1) $$A_{w}=\frac{1}{1000}<\text{CT}{>}_{\text{ROI}}A_{\mathrm{ROI}}+A_{\mathrm{ROI}}$$

The air surrounding the object should have negligible impact on the result.^16^ To account for the attenuation of the CT table, *A*_w_ (table) was determined by manually contouring the table (ROI__table_) in one CT image for each scanner, and then substituting A_ROI_table_ and <CT>_ROI_table_ in eq. (1).

For each examination included in the study, a complete sequence of CT images (image series) was downloaded from the PACS and analyzed using ImageJ software (National Institute of Health, Bethesda, MA, USA), with a macro written by the authors. For each CT image, this macro extracted the values of table position (*z*) and tube current (*I*_*z*_) from the DICOM header, then drew a region of interest (ROI) encompassing the entire FOV, and determined its area (*A*_ROI_) and mean CT number (<CT>_ROI_). The results were transferred to a spreadsheet, and *D*_w_(*z*) was calculated using eq. (2), 16:

(2) $$D_{w}\left(z\right)=2\sqrt{\frac{A_{w}\left(z\right)-A_{w}\left(\text{table}\right)}{\pi }}$$

The automated method to obtain *D*_w_ was tested using two cylindrical acrylic phantoms (32 cm and 24 cm in diameter), filled with water, and imaged with the clinical protocol for chest. The *D*_w_ results obtained were in good agreement (better than 0.2 cm) with expected values.

The values of *D*_w_ obtained with the automated method were compared with *D*_w_ obtained from manual patient contouring in two images (one in the thorax and one in the abdomen) for a total of ten examinations (five in CT11 and five in CT14).

The values of *D*_w_(*z*) obtained from patient images were used to calculate two different quantities: the mid‐scan or central *D*_w_c_, which is the value of *D*_w_(*z*) in the middle of the scanned region; and the *D*_w_ave_, calculated as the mean of all *D*_w_(*z*) values in the imaged region. *D*_w_c_ and *D*_w_ave_ were determined for all examinations, and SSDE was calculated as CTDI_vol_ × f (*D*_w_c_), where f (*D*_w_c_) is the conversion factor related to a patient diameter of *D*_w_c_, obtained from AAPM tables.^15^, ^16^

### 2.C Mathematically simulated scenarios

To simulate acquisitions with different values of *I*_min_, the original values of tube current were thresholded in the spreadsheet, so that all *I*(*z*) values below a certain *I*_min_ were made equal to that *I*_min_. The mean of *I*(*z*) was calculated for each simulated scenario, and the corresponding examination CTDI_vol_ determined. Simulated values of *I*_min_ were 210 mA and 280 mA for CT11 and 140 mA and 180 mA for CT14.

This approach has some limitations, because only values of *I*_min_ higher than the original can be simulated. Moreover, it assumes a mere cutoff at the limiting value. Preliminary phantom measurements (B. Mendes, F. Dias, D. Oliveira, S. Sarmento, unpublished data) suggest that real ATCM systems are more sophisticated and adjust spatial variations according to the intended range. But the resulting difference between real and simulated CTDI_vol_ was found to be less than 5% for these two scanners.

## 3 RESULTS

### 3.A Dosimetric plots as a function of different metrics

**Figure 1:**
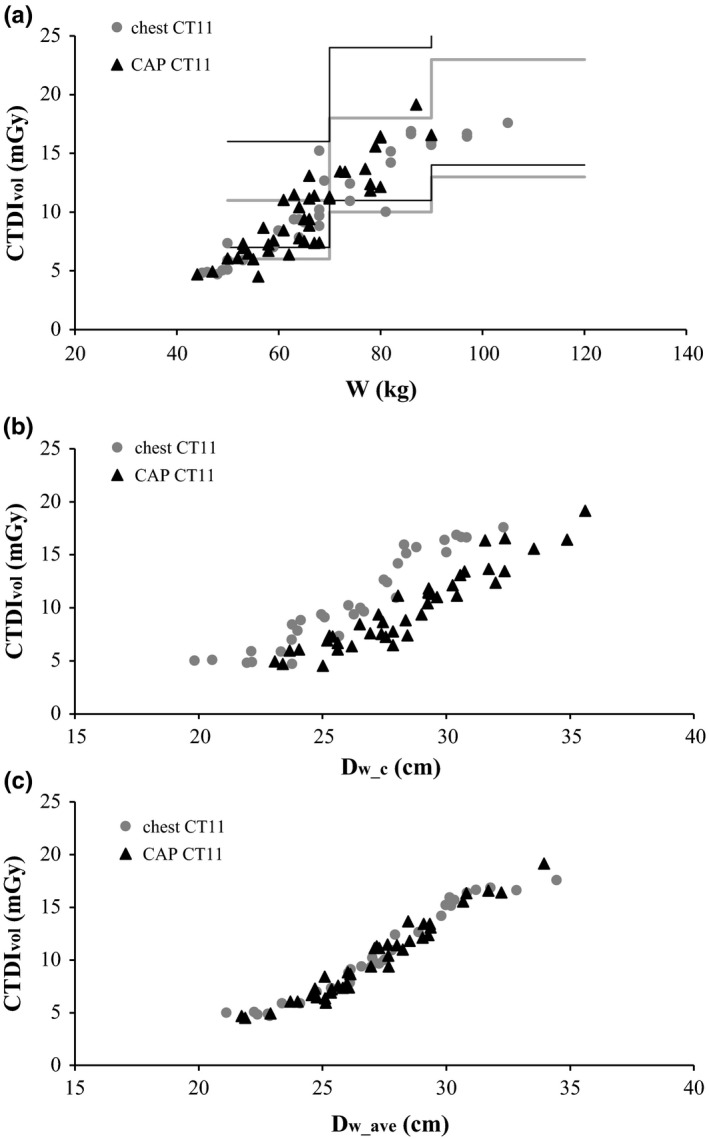
CTDI_vol_ as a function of patient weight (a), D_w_c_ (b), and D_w_ave_ (c), for chest and CAP examinations performed in the CT11 scanner. AAPM reference values^12^, ^13^ are shown as horizontal/vertical lines (gray — chest and black — CAP).

**Figure 2:**
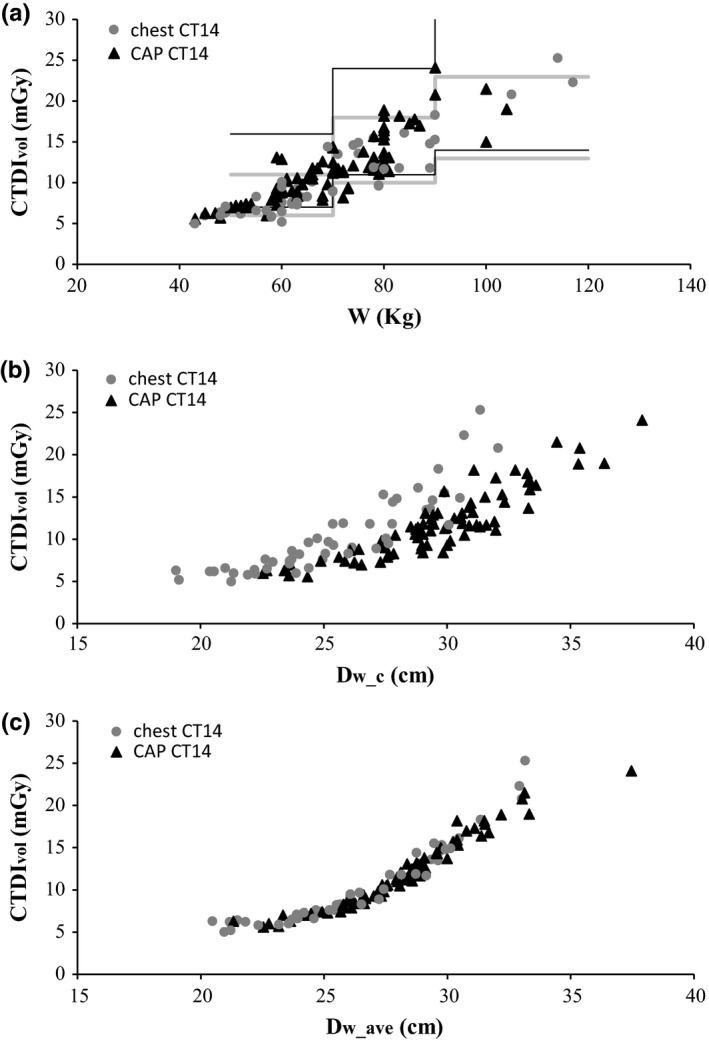
CTDI_vol_ as a function of patient weight (a), D_w_c_ (b), and D_w_ave_ (c), for chest and CAP examinations performed in the CT14 scanner. AAPM reference values^12^, ^13^ are shown as horizontal/vertical lines (gray — chest and black — CAP).

Values of CTDI_vol_ are plotted as a function of patient weight (a), *D*_w_c_ (b), and *D*_w_ave_ (c) in Figs. 1 and 2, for chest and CAP examinations performed in CT11 and CT14, respectively.

The approximate CTDI_vol_ values for different weight ranges listed in AAPM protocols are shown for comparison.^12^, ^13^ The dispersion of CTDI_vol_ data in Figs. 1(a) and 2(a) reflects the different heights of patients with similar weight, as well as different mass distributions in the body. When CTDI_vol_ is plotted as a function of *D*_w_c,_ in Figs. 1(b) and 2(b), there is a separation of data for chest and CAP examinations, related to the different anatomical location of the mid‐scan slice. *D*_w_c_ is obtained in the middle of the lungs (low attenuation region) in chest examinations and closer to the liver in CAP examinations.

Plotting CTDI_vol_ as a function of *D*_w_ave_ reduces data dispersion to a minimum, as shown in Figs. 1(c) and 2(c). Details become clearer, like the flattening of the curves for very small and very large patient sizes, which is probably related to *I*_min_ and *I*_max_. As expected, the dependence of CTDI_vol_ on *D*_w_ave_ is the same for CAP and chest examinations — these examinations are performed with the same ATCM settings, in both scanners.

**Figure 3:**
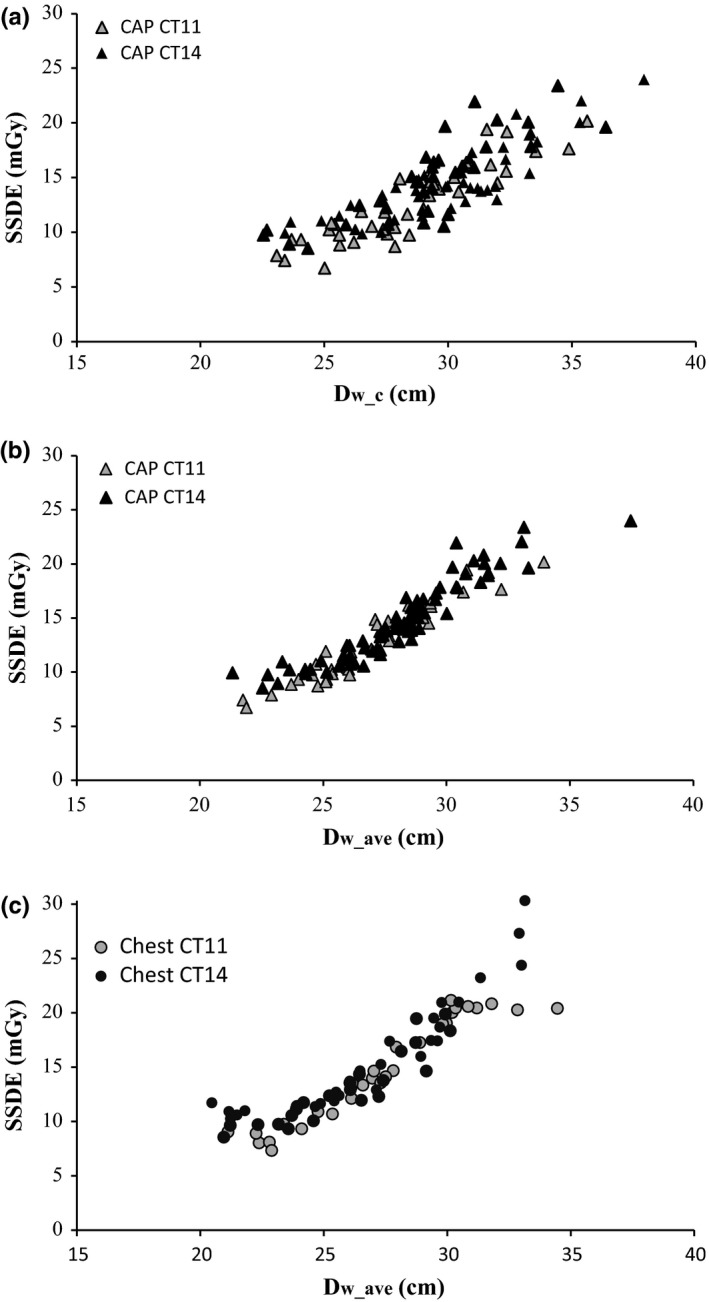
SSDE plotted as a function of D_w_c_ (a) for CAP examinations and as a function of D_w_ave_ for CAP (b) and chest (c) examinations, for both CT scanners.

SSDE values are plotted in Fig. 3(a) as a function of *D*_w_c_ (CAP examinations) and as a function of *D*_w_ave_ in Fig. 3(b) (CAP) and 3(c) (chest). The use of *D*_w_ave_ reduces data dispersion in plots of SSDE, as it did for CTDI_vol_. To allow a more quantitative comparison, *R*‐squared values are presented in Table 4 for linear fits to CTDI_vol_ and SSDE data for CAP examinations.

**Table 4:** *R*‐squared values obtained for linear fits to CAP CTDI_vol_ and SSDE values vs. different patient size metrics

	*R*‐squared values for linear fits			
	CTDIvol		SSDE	
	CT14	CT11	CT14	CT11
Weight	0.72	0.80	0.68	0.78
BMI	0.73	0.78	0.73	0.76
*D*_w_c_	0.81	0.89	0.69	0.80
*D*_w_ave_	0.93	0.94	0.87	0.92

Before the widespread use of ATCM systems, Menke tested different surrogates for mean patient attenuation and concluded that the correlation between patient attenuation and body mass index (BMI) was no better than with patient weight.^19^ A similar result was obtained in this study, as shown in Table 4.

### 3.B Mathematically simulated scenarios

**Figure 4:**
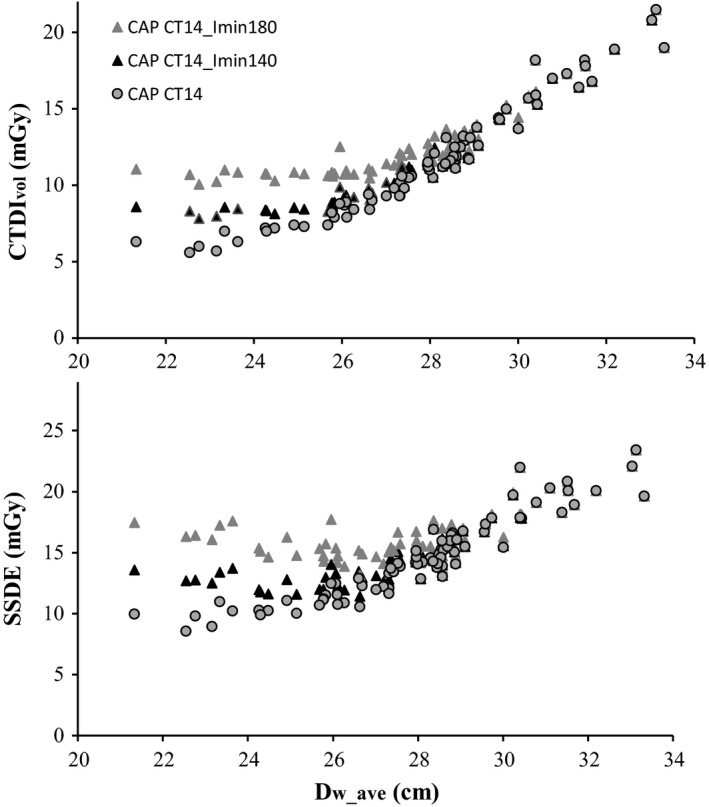
CTDI_vol_ and SSDE plotted as a function of D_w_ave_ for the mathematically simulated CAP examinations in CT14 with I_min_ values of 140 mA and 180 mA. The original dosimetric data from the CAP examinations are plotted in the same graphs for comparison.

In Fig. 4, CTDI_vol_ and SSDE are plotted as a function of *D*_w_ave_, for the original dosimetric data in CT14 and for the mathematically simulated CAP examinations with *I*_min_ values of 140 mA and 180 mA. When *I*_min_ is set at 180 mA, CTDI_vol_ remains approximately constant for small patients (*D*_w_ave_<25 cm) and then increases gradually until, for large patients (*D*_w_ave_ ≈ 30 cm), it reaches the values obtained at lower *I*_min_ settings. As a result, a high value of *I*_min_ reduces the range of variation of CTDI_vol_ with patient size. A similar effect is observed for SSDE (Fig. 4).

The mean values and standard deviation (SD) of CTDI_vol_ and SSDE are presented in Table 5, for the real examinations and for the simulated scenarios.

**Table 5:** Statistics of CTDI_vol_ and SSDE, presented as mean values ± standard deviations, for the real examinations and the simulated scenarios

	CTDI_vol_ (mGy)	SSDE (mGy)
CT14	11.8 ± 4.0	14.6 ± 3.6
CT14_Imin140	12.2 ± 3.5	15.2 ± 3.0
CT14_Imin180	13.1 ± 2.9	16.5 ± 2.3

### 3.C Interdependence of different metrics

The patient sample considered in this work is representative of a particular population of oncological patients (Tables 1 and 2). Mean male and female heights agree well with known statistics for the Portuguese population in this age‐group.^24^ The data obtained in this work were compared with the earlier study by Menke of a different patient population, aged 18–87 years, with weight 46–108 kg, height 153–200 cm (mean 170 cm), and BMI 16–38 kg/m^2.^ 19

**Figure 5:**
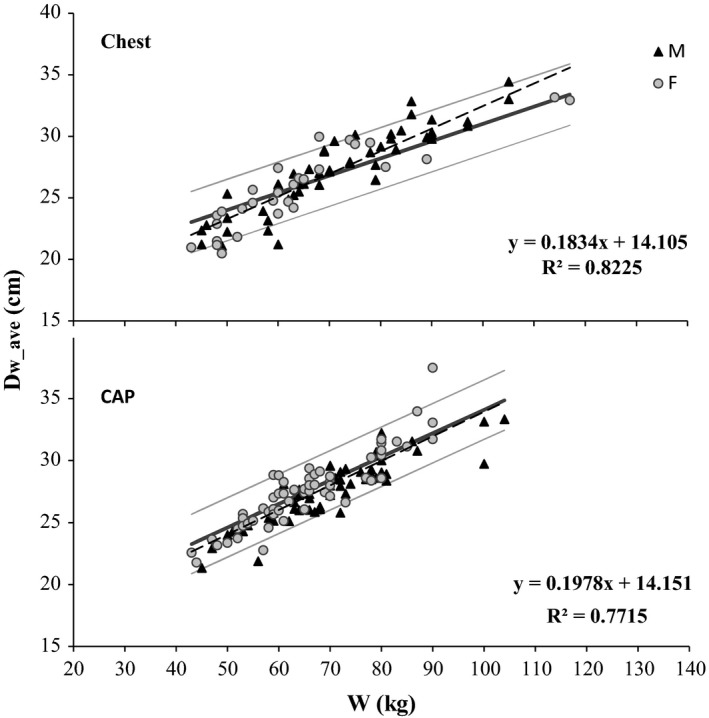
D_w_ave_ plotted as a function of patient weight, for chest and CAP examinations. The broken lines represent the regression equations obtained for our own data, without separation of male and female patients (equations shown above), while the light gray lines represent the regression equation (thick middle line) and the 95% prediction limits (outer thin lines) obtained by Menke for chest and abdominal examinations, respectively.^19^

In Fig. 5, *D*_w_ave_ is plotted as a function of patient weight for chest and CAP examinations. The correlations found by Menke for chest and abdominal examinations are shown for comparison. For chest CT, the data from this study fall mostly within the 95% prediction limits previously obtained for this unrelated population ^19^ (Fig. 5), despite the difference in the obtained regression equation. It is unclear whether this difference results from intrinsic population metrics, or there is some additional bias in this study due to oncological risk factors and/or effect of oncological treatments.

For CAP examinations, the variation of *D*_w_ave_ with patient weight is similar to that obtained by Menke for abdominal examinations (Fig. 5).

**Figure 6:**
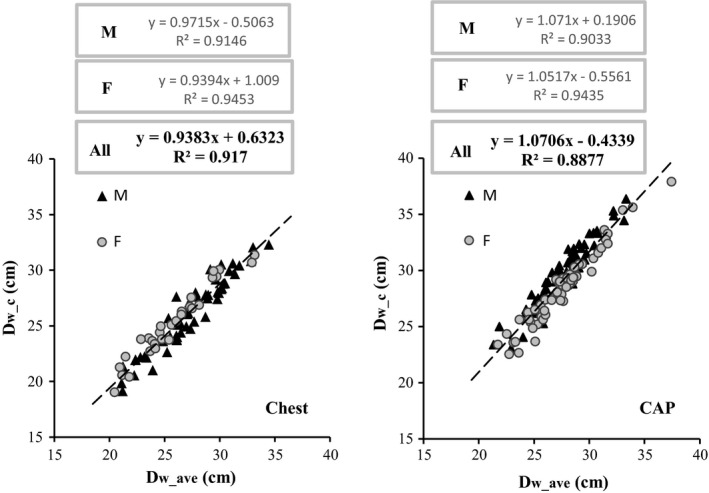
D_w_c_ plotted as a function of D_w_ave_ for chest and CAP examinations. The dashed lines represent the linear fits to all the data (male and female patients). Fits to the two subsets (male and female) are shown only as equations.

**Figure 7:**
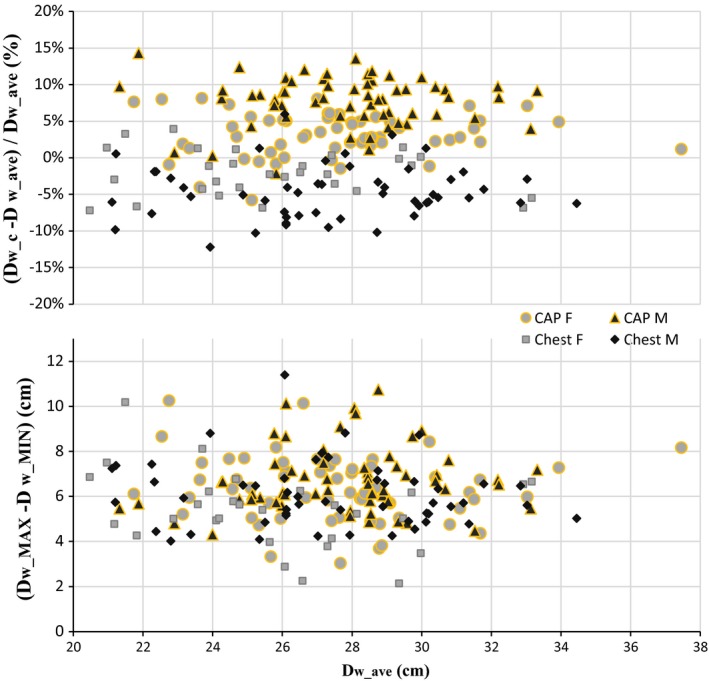
Percentage difference between D_w_c_ and D_w_ave_ (above) and maximum absolute D_w_ variation (below), plotted as a function of D_w_ave_, for all examinations and patients in the study.

There is good correlation between *D*_w_c_ and *D*_w_ave,_ for both examination types studied, as shown in Fig. 6. The two metrics are very similar, with maximum percentage differences below 15% (in % of *D*_w_ave_) as shown in Fig. 7 and summarized quantitatively in Table 6. There appears to be some separation of male and female patients, probably related to differences in body habitus. The mean *D*_w_c_ − *D*_w_ave_ difference for chest CT was −4 ± 4% (in % of *D*_w_ave_), which is comparable with the −1 ± 4% reported by Anam et al.^20^

**Table 6:** Differences between *D*_w_c_ − *D*_w_ave_ and *D*_w_(max) −* D*_w_(min), represented as mean ± standard deviation, for different examinations and patient groups (see plots in Fig. 7)

		*D*_w_c_ − *D*_w_ave_		*D*_w_(max) − *D*_w_(min)	
		(% of *D*_w_ave_)	(cm)	(% of *D*_w_ave_)	(cm)
Chest	F	−2 ± 3	−0.6 ± 0.8	22 ± 9	5.4 ± 1.7
	M	−5 ± 4	−1.3 ± 1.0	23 ± 7	6.1 ± 1.5
	All	−4 ± 4	−1.0 ± 1.0	22 ± 7	5.8 ± 1.6
CAP	F	+3 ± 3	+0.9 ± 0.8	23 ± 7	6.4 ± 1.5
	M	+8 ± 3	+2.2 ± 0.9	24 ± 5	6.7 ± 1.4
	All	+5 ± 4	+1.5 ± 1.1	24 ± 6	6.5 ± 1.4

The variation of *D*_w_ found in each examination, *D*_w___max_ − *D*_w___min_, is similar for male and female patients, as shown in Fig. 7 and Table 6. The values agree well with those reported by Leng et al. for a different population (weight 37–183 kg, mean 85 kg, BMI 15–57 kg/m^2^, mean 29 kg/m^2^), where *D*_w_ variation was 5.2 ± 1.4 cm for chest and 6.5 ± 1.3 cm for CAP examinations, and maximum *D*_w___max_ − *D*_w___min_ was 10.5 cm (32% of *D*_w_ave_).^21^ In this study, *D*_w_ variation was 5.8 ± 1.6 cm for chest and 6.5 ± 1.4 cm for CAP examinations (Table 6).

### 3.D Feasibility of small‐scale studies in nonautomated scenarios

At our institution, *D*_w_ave_ proved easier to obtain than patient weight. Recording patient biometric data requires technologist time and interferes with workflow, while downloading images from PACS may be carried out by medical physicists elsewhere. Using *D*_w_ave_ also allowed retrospective studies and comparisons, for all CT examinations where full FOV images were available.

## 4 ANALYSIS AND DISCUSSION

### 4.A Dosimetric plots as a function of different metrics

Despite the very similar values of *D*_w_c_ and *D*_w_ave_, total examination doses (CTDI_vol_ or SSDE) clearly have a stronger correlation with *D*_w_ave_ than with *D*_w_c_, as reflected by the lower dispersion of dosimetric data (Figs. 1, 2, and 3, Table 4). The values of *D*_w_c_ probably reflect both variations in scan length (which alter the anatomical location of the midscan slice) and localized anatomy characteristics like abdominal obesity, or large breasts in some female patients. This makes *D*_w_ave_ the most advantageous metric for the purpose of protocol optimization and automatic detection of outliers or accidental changes, despite the additional computational effort involved in its calculation.

Another advantage of *D*_w_ave_ is the similar dependence of CTDI_vol_ and SSDE for both examination types (Figs. 1 and 2). Identifying examination type can be challenging for automatic systems, because examination and protocol nomenclature are rarely standardized. Moreover, some examinations have more than one sequence (e.g., before and after contrast injection), making it difficult to compare total DLP.^25^ Our data suggest that comparing CTDI_vol_ vs. *D*_w_ave_ for a group of different examinations (performed with similar protocols) may be a feasible alternative in some situations.

### 4.B Mathematically simulated scenarios

The mathematically simulated scenario with *I*_min_ = 180 mA illustrates how patient doses may increase by nearly 50% for adults under 70 kg, while mean examination doses increase by only 12–13%, and maximum values are not exceeded (Fig. 4 and Table 5). This highlights the importance of establishing diagnostic reference levels as a function of patient size, to allow quick detection of nonoptimized protocols by dose monitoring software. Comparison with established references could also be made before irradiation, if *D*_w_ were determined from scout images as suggested by TG220.^16^

It is not the purpose of this work to determine ideal dose levels for small and large adults, or discuss whether CTDI_vol_ should increase linearly with patient size. For adult patients, optimum variation of examination dose with size remains a matter of debate. Noise constant systems such as GE and Toshiba result in a linear increase in CTDI_vol_ with patient weight, but some ATCM systems intentionally decrease dose less for thinner adults.^26^ A comparison of CT scanners from three manufacturers showed that the Philips system had the least variation of DLP with patient weight, when compared with GE and Siemens.^27^ Some authors using GE scanners divide adult patients into weight categories.^7^

More data are necessary, especially as automatic selection of tube voltage may soon be a widespread option as well.^28^ The example presented here merely highlights the importance of choosing a patient size metric which reduces data dispersion to a minimum, to improve detection of normal trends and outliers.

### 4.C Interdependence of different metrics

The comparisons shown in Fig. 5 are an encouraging result, suggesting that the study of a sufficiently large number of different populations and anatomical regions might provide a conversion between patient weight and *D*_w_ave_, for each examination type. This would allow comparison of newer large‐scale data based on *D*_w_ metrics with the existing studies and standards based on patient weight.

As reported by other authors, the impact of *D*_w_ave_ − *D*_w_c_ differences on SSDE values is small,^21^ because the two metrics have quite similar values. This is reflected in the small data dispersion seen in Fig. 3(b) and 3(c). However, the lower dispersion of dosimetric data when *D*_w_ave_ is used as a metric for patient size suggests that the effect of small *D*_w_ave_ − *D*_w_c_ differences is probably amplified by the large variation in CTDI_vol_ (from ~8 mGy to ~16 mGy, a nearly 100% increase) which occurs for a relatively small increase in *D*_w_ave_ (from ~25 cm to ~31 cm, ~24% increase), as shown in Figs. 1(c) and 2(c). The effect may be less pronounced when using ATCM systems from different vendors.

These results highlight the importance of automatic calculation of *D*_w_ave_, despite the additional computational effort involved. Moreover, these data suggest that *D*_w_ave_ needs to be determined with great accuracy and in a standardized manner, if it is to be used for comparisons between different CT scanners and different institutions. In this work, the mean difference between automated and manual *D*_w_ was found to be 0.2 ± 1.2% (0.05 ± 0.32 cm), but the mean absolute difference was 1.0 ± 0.7% (0.26 ± 0.19 cm), with a maximum difference of 2.2% (0.6 cm).

Drawing a ROI including the entire FOV is computationally fast and simple. But the greater accuracy (maximum 0.5% difference) reported for automatic contouring^20^ should prove useful for dose comparisons, particularly during initial studies and acquisition of baseline data.

## 5 CONCLUSIONS

This study highlights the importance of automatic calculation of *D*_w_(*z*), not just for organ dose estimation as already recommended,^16^, ^18^ but also to make *D*_w_ave_ available to end users as a patient size metric for binning similar‐sized patients in RDIM systems.

Despite the small percentage difference between *D*_w_c_ and *D*_w_ave_ (−4 ± 4% for chest and +5 ± 4% for CAP examinations in this study), both CTDI_vol_ and SSDE present a stronger correlation with *D*_w_ave_ than they do with *D*_w_c_. Our data suggest *D*_w_c_ values reflect localized anatomy characteristics. The lower dispersion of dosimetric data obtained with *D*_w_ave_ makes it easier to identify trends and outliers. This is useful for ATCM optimization and detection of accidental changes. Use of *D*_w_ave_ also reduces dependence on examination type, which may be difficult to identify accurately in large‐scale databases. Therefore, when implementing automatic determination of *D*_w_c_ for SSDE calculations, automatic calculation of *D*_w_ave_ should definitely be considered as well, despite the additional computational effort involved.

Use of *D*_w_ metrics is not yet widely implemented in CT scanners and RDIM systems, but it is important to acquire baseline data for *D*_w_ave_ metrics and to establish comparisons with existing standards based on patient weight. Our experience shows that small‐scale studies using *D*_w_ave_ metrics are feasible in nonautomated scenarios and may be used initially to acquire baseline data from new and retrospective studies.

## CONFLICT OF INTEREST

The authors declare that they have no conflict of interests.

## References

[1] Sutton D , McVey S , Gentle D . CT chest abdomen pelvis doses in Scotland: has the DRL had its day? Br J Radiol. 2014;87:20140157. 24971617 10.1259/bjr.20140157 PMC4453148

[2] McCollough C , Branham T , Herlihy V , et al. Diagnostic reference levels from the ACR CT accreditation program. J Am Coll Radiol. 2011;8:795–803. 22051465 10.1016/j.jacr.2011.03.014

[3] European Commission . Radiation Protection 109 Guidance on Diagnostic Reference Levels (DRLs) for Medical Exposures; 1999.

[4] Shrimpton PC , Hillier MC , Meeson S , Golding SJ . PHE‐CRCE‐013 – Doses from Computed Tomography (CT) Examinations in the UK – 2011 Review; 2011.

[5] Kalra MK , Maher MM , Toth TL , Schmidt B , Westerman BL , Morgan HT . Radiology techniques and applications of automatic tube current modulation for CT. Radiology. 2004;233:649–657. 15498896 10.1148/radiol.2333031150

[6] Kalra MK , Maher MM , Kamath RS , et al. Sixteen‐detector row CT of abdomen and pelvis: study for optimization of Z‐axis modulation technique performed in 153 patients. Radiology. 2004;233:241–249. 15454622 10.1148/radiol.2331031505

[7] Goldman AR , Maldjian PD . Reducing radiation dose in body CT: a practical approach to optimizing ct protocols. AJR Am J Roentgenol. 2013;200:748–754. 23521442 10.2214/AJR.12.10330

[8] McCollough CH , Bruesewitz MR , Kofler JM . CT dose reduction and dose management tools: overview of available options. RadioGraphics. 2006;26:503–513. 16549613 10.1148/rg.262055138

[9] Söderberg M , Gunnarson M . Automatic exposure control in computed tomography – an evaluation of systems from different manufacturers. Acta radiol. 2010;51:625–634. 20429764 10.3109/02841851003698206

[10] Yu L , Bruesewitz MR , Thomas KB , Fletcher JG , Kofler JM , Mccollough CH . Optimal tube potential for radiation dose reduction in pediatric CT: principles, clinical implementations, and pitfalls. Radiographics. 2011;31:835–848. 21571660 10.1148/rg.313105079

[11] Kleinman PL , Strauss KJ , Zurakowski D , Buckley KS , Taylor GA . Patient size measured on CT images as a function of age at a tertiary care children's hospital Patricia. Am J Roentgenol. 2010;194:1611–1619. 20489103 10.2214/AJR.09.3771

[12] American Association of Physicists in Medicine (AAPM) . Working Group on Standardization of CT Nomenclature and Protocols: Adult Routine Chest CT protocols Version 1.0 11/20/2012; 2012. Available at: http://www.aapm.org/pubs/CTProtocols/documents/AdultRoutineChestCT.pdf. Accessed August 14; 2015.

[13] American Association of Physicists in Medicine (AAPM) . Working Group on Standardization of CT Nomenclature and Protocols: Adult Routine Chest‐Abdomen‐Pelvis CT protocols Version 1.0 02/20/14; 2014 Available at: http://www.aapm.org/pubs/CTProtocols/documents/AdultRoutineChestAbdomenPelvisCT.pdf. Accessed August 14, 2015.

[14] Gress DA , Dickinson RL , Erwin WD , et al. AAPM medical physics practice guideline 6.a.: performance characteristics of radiation dose index monitoring systems. J Appl Clin Med Phys. 2017. 10.1002/acm2.12089 PMC5875816 28497529

[15] American Association of Physicists in Medicine (AAPM) . Size‐Specific Dose Estimates (SSDE) in Pediatric and Adult Body CT Examinations – Report of AAPM Task Group 204; 2011.

[16] American Association of Physicists in Medicine (AAPM) . Use of Water equivalent diameter for calculating patient size and size‐specific dose estimates (SSDE) in CT – Report of AAPM Task Group 220. College Park, MD: American Association of Physicists in Medicine ; 2014. PMC4991550 27546949

[17] Christner JA , Braun NN , Jacobsen MC , Carter RE , Kofler JM , Mccollough CH . Size‐specific dose estimates for adult patients at CT of the torso. Radiology. 2012;265:841–847. 23091173 10.1148/radiol.12112365

[18] Bostani M , McMillan K , Lu P , et al. Attenuation‐based size metric for estimating organ dose to patients undergoing tube current modulated CT exams. Med Phys. 2015;42:958. 25652508 10.1118/1.4906132 PMC6961670

[19] Menke J . Comparison of different body size parameters for individual dose adaptation in body CT of adults. Radiology. 2005;236:565–571. 16040914 10.1148/radiol.2362041327

[20] Anam C , Haryanto F , Widita R , Arif I , Dougherty G . Automated calculation of water‐equivalent diameter (D W) based on AAPM Task Group 220. J Appl Clin Med Phys. 2016;17:320–333. 10.1120/jacmp.v17i4.6171 PMC5690059 27455491

[21] Leng S , Shiung M , Duan X , Yu L , Zhang Y , Mccollough CH . Size‐specific dose estimates for chest, abdominal, and pelvic CT: effect of intrapatient variability in water‐equivalent diameter. Radiology. 2015;276:184–190. 25734556 10.1148/radiol.15142160 PMC4479973

[22] Sociedad Española de Física Médica . Protocolo Español de Control de Calidad En Radiodiagnóstico. Madrid: Sociedad Española de Física Médica; 2011.

[23] Ikuta I , Warden GI , Andriole KP , Khorasani R , Sodickson A . Estimating patient dose from x‐ray tube output metrics: automated measurement of patient size from CT images enables large‐scale size‐specific dose estimates. Radiology. 2014;270:472–80. 24086075 10.1148/radiol.13122727 PMC4228751

[24] Organisation for Economic Co‐Operation and Development (OECD) . Society at a Glance 2009 – OECD Social Indicators. Organisation for economic co‐operation and development (OECD); 2009 https://doi.org/10.5860/choice.47-2382.

[25] Nicol RM , Wayte SC , Bridges AJ , Koller CJ . Experiences of using a commercial dose management system (GE DoseWatch) for CT examinations. Br J Radiol. 2015;2016:20150617. 10.1259/bjr.20150617 PMC12369700 26539632

[26] Rizzo S , Kalra M , Schmidt B , et al. Comparison of angular and combined automatic tube current modulation techniques with constant tube current CT of the abdomen and pelvis. Am J Roentgenol. 2006;186:673–679. 16498094 10.2214/AJR.04.1513

[27] Iball G , Tout D . Computed tomography automatic exposure control techniques in 18F‐FDG oncology PET‐CT scanning. Nucl Med Commun. 2014;35:372–281. 24445670 10.1097/MNM.0000000000000064

[28] Lee KH , Lee JM , Moon SK , et al. Attenuation‐based automatic tube voltage selection and tube current modulation for dose reduction at contrast‐enhanced liver CT. Radiology. 2012;265:437–447. 23012467 10.1148/radiol.12112434

